# Unveiling earthquakes: thermoluminescence signal resetting of a natural polymineral sample in laboratory-produced fault gouge

**DOI:** 10.1038/s41598-026-47125-1

**Published:** 2026-04-19

**Authors:** Maryam Heydari, Sebastian Kreutzer, Chien-Cheng Hung, Mohammad R. Ghassemi, Loïc Martin, Sumiko Tsukamoto, Frank Preusser, André Niemeijer

**Affiliations:** 1 Institute of Earth and Environmental Sciences, University of Freiburg, Albertstr. 23B, 79104 Freiburg, Germany; 2 Institute of Geography, Heidelberg University, Im Neuenheimer Feld 348, 69120 Heidelberg, Germany; 3 LIAG Institute for Applied Geophysics, Stilleweg 2, 30655 Hanover, Germany; 4 HPT Laboratory, Department of Earth Sciences, Utrecht University, Utrecht, The Netherlands; 5 Research Institute for Earth Sciences, Geological Survey of Iran, Azadi Square, Meraj Avenue, P.O. Box 131851494, Tehran, Iran; 6 Institute for Geology, University of Innsbruck, Innsbruck, Austria; 7 Department of Geosciences, University of Tübingen, Schnarrenbergstr. 94-96, 72076 Tübingen, Germany

**Keywords:** Natural hazards, Solid Earth sciences

## Abstract

Unravelling a reliable timing of past earthquakes through luminescence dating of fault gouge depends on sufficient frictional heat during a co-seismic event to fully reset the luminescence signal. Laboratory fault-gouge production using a rotary shear apparatus has attracted interest as a method for probing the degree of signal resetting in quartz during friction experiments. However, natural fault gouges are complex, exhibiting a mixture of minerals that are specific to the host rocks. Here, we employed a host rock sample from the Alborz Mountains (Iran) and subjected it to a friction experiment without any chemical treatment, after being reset and irradiated with a known gamma dose. We performed a medium-slip velocity friction experiment with a slip velocity of 0.05 m/s and a normal stress of 12 MPa, while the temperature evolution of the gouge zone was recorded using an infrared camera. The thermographic images show a transient temperature of approximately 296 °C, with the luminescence signal resetting at a small, extremely localised slip patch, confirming the challenges involved in identifying the best spot for signal resetting. However, we identified a high-temperature signal enhancement in the thermoluminescence (TL) curves that might serve as a marker for fault-gouge formation.

## Introduction

Unravelling the timing of the most recent seismic activity of faults in tectonically active regions is pivotal for our understanding of temporal fault movements and developing effective geohazard mitigation strategies. While the most recent activity of faults during the instrumental period can be readily recorded, the covered period is limited, leaving earthquakes with longer recurrence intervals spanning a few hundred years to millennia unmonitored. In those cases, markers on the Earth’s surface formed during major earthquakes, known as deformed geomorphic and sedimentary features, are studied. Studying these markers, combined with geochronological methods, can provide insight into fault activity on timescales beyond instrumental records^1^. Aside from probing surface deformations, in the upper crust, different types of fault rocks are formed due to fault movements in conjunction with a suite of geochemical processes^2^. Among various fault rocks, fault gouge, the product of abrasive wear, stands out as it is produced at shallow depth. As such, radiometric dating of newly formed clay minerals in fault gouge has been used to decipher the brittle deformation history of the upper crust^3^.

Recently, dosimetric dating methods have attracted increasing attention in palaeoseismology because of their low closure temperature, which may provide information on the timing of the most recent fault activity^4^. For instance, trapped charge dating methods, including luminescence dating, have been widely applied to fault gouges to determine the faults’ most recent activity. However, studies worldwide indicate that the obtained values yield overestimated ages in several cases^4–7^. In other studies, accurate estimates of the timing of past fault movement were obtained using trapped charge dating methods^8–10^. Thus, it appears that this technique is susceptible to the particular regional and co-seismic event setting, for instance, host rock composition, slip velocity, and shear stress. One of the recent attempts to constrain the timing of the most recent earthquakes used luminescence dating at the North Tehran Fault (NTF) in the Southern Alborz Mountains^11,12^. These ages are interpreted as overestimates in light of data from the historical earthquake catalogue. Notably, the dating results showed a correlation between the sampling location in the exhumed fault rocks and the determined ages^12^. This observation suggests that the signal resetting due to frictional heat during a major earthquake is not uniformly distributed within the fault gouge and that the sampling location significantly influences the age results. Using a laboratory-controlled friction experiment, Kim et al.^13^ demonstrated that frictional heat production and dissipation vary within fault thickness.

As such, sufficient knowledge of the generated frictional heat and its effects on the fault rock is vital for understanding the degree of signal resetting and for obtaining reliable ages of the most recent seismic slip through fault-gouge dating.

Friction experiments using a rotary shear apparatus simulate slip of fault gouges in the laboratory under controlled conditions. The resulting experimental products can then be used to probe the degree of signal resetting. One recent study, conducted by Yang et al.^14^, found that trapped-charge dating methods for irradiated quartz fully reset samples when a normal stress of 1 MPa and a slip velocity of 2 m/s were applied. Kim et al.^13^ reduced the slip velocity to 1.31 m/s and indicated that the probed optically stimulated luminescence (OSL,^15^) signal from the localised slip layer of quartz was fully reset. Oohashi et al.^16^ demonstrated that even with slip velocities slower than 1.31 m/s but faster than 0.65 m/s, the quartz OSL signal is completely depleted. Older studies (limestone: Zeller et al.^17^, quartz^18^) investigated the effect of pressure on the thermoluminescence (TL) signal. However, while this may indeed cause changes in the emission characteristics at high pressure (> 1 GPa), the effects appear negligible compared to friction-induced heating.

The three main parameters controlling the temperature rise in a friction experiment are shear stress (determined by the applied normal stress through the coefficient of friction), slip duration, and slip velocity. However, the thermodynamic properties of the rock of interest (e.g., density, heat capacity, and thermal diffusivity) also influence temperature evolution during friction experiments^2,19^. The hydrostatic-pressure experiment by Zöller et al.^20^ revealed that pressure alone may not be sufficient to reset thermoluminescence (TL) signals. In particular, clay minerals can significantly alter the friction parameters when their contribution to the overall material is not negligible. However, their role in resetting the luminescence signal is poorly understood. To get a better grasp of the temperature rise in nature and the potential luminescence signal resetting, it seems, therefore, crucial to use the host rock of the fault of interest during the friction experiment.

In this study, we use an intact, chemically untreated host rock to produce fault gouges in the laboratory via a rotary shear experiment. This generally follows previous friction studies^13,14,16^, however, instead of quartz extracts, we investigate the signal resetting of a polymineral fraction of a host rock. Although slip velocities during earthquakes can reach 10 m/s^21,22^, our experiments were performed at 0.05 m/s, the limit of our apparatus. Since an earlier attempt found that a combination of a slip velocity below 0.06 m/s and a normal stress of 1 MPa resulted in a non-resetting signal^13^, we increased the normal stress to 12 MPa to compensate for the low slip velocity. This setting enabled us to address whether increasing the normal stress, rather than the slip velocity, can lead to full or partial signal resetting at a minimum. During friction experiments, we use a high-speed infrared camera to monitor the spatial and temporal evolution of the gouge zone temperature.

Additionally, we examine the TL signal change from the polymineral fine-grain fraction as a direct measure of the signal resetting of the produced fault gouge. We will discuss our findings from the polymineral TL signal.

## Materials and methods

**Fig. 1:**
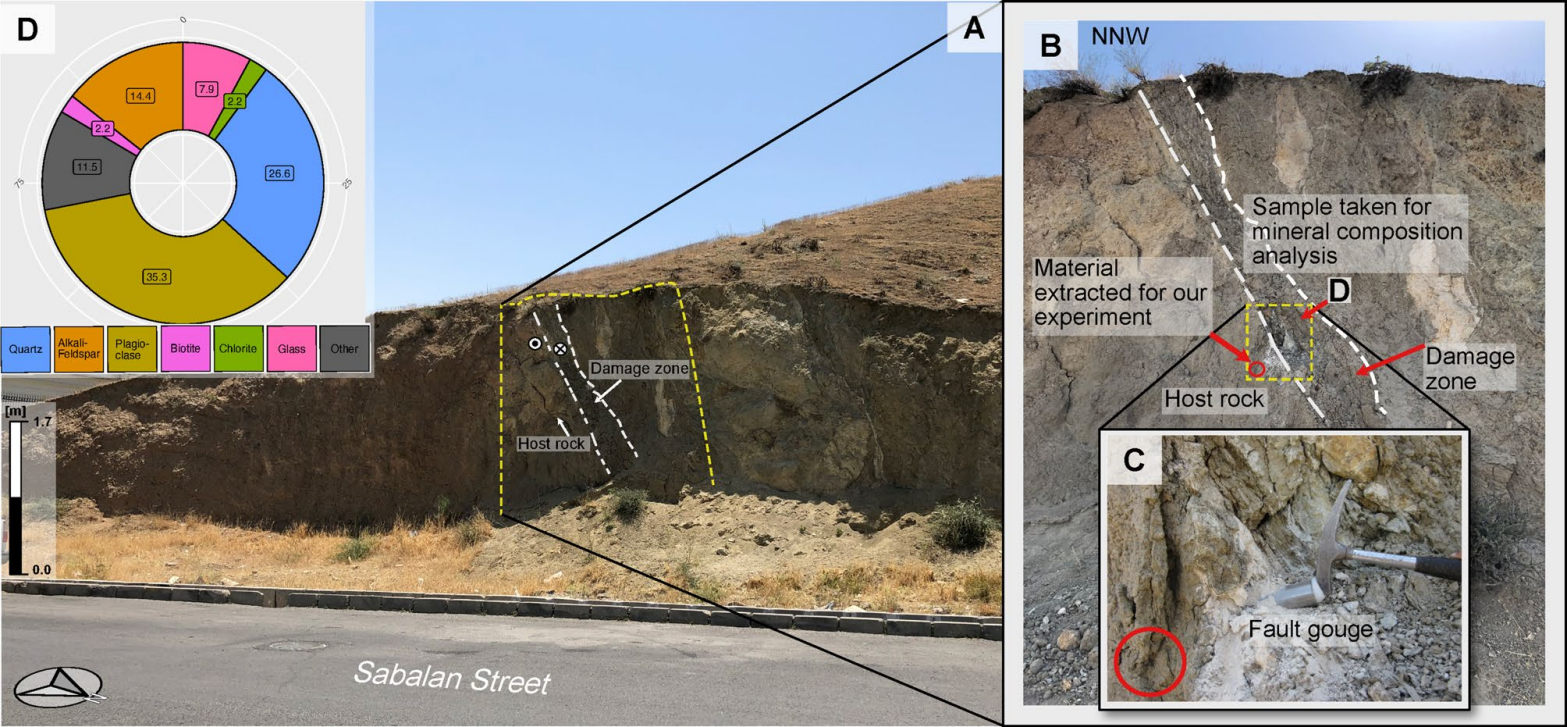
Sampling location at the fault rock outcrop of the secondary branch of the NTF zone in the south of the Alborz Mountains. (**A**) The location of the rock sample extracted for the friction experiment is marked with a red circle in (**B**) and (**C**). The damage zone and produced fault gouge due to the activity of the NTF are marked in insets (**B**) and (**C**). (**D**) Mineral composition of the sample close to the extracted material for the friction experiment derived from SEM measurements.

Our sample originates from the southern front of the Alborz Mountains and was extracted from the host rock of one of the secondary branches of the NTF zone, where fault movement is associated with strike-slip motion^23,24^. We sampled the host rock close to the fault gouge, which is identified by its white colour (Fig. 1). The host rock is composed of Eocene volcaniclastic rocks known as the Karaj Formation^25,26^. Scanning Electron Microscopy (SEM; JEOL FEG-SEM JSM-IT800) equipped with an electron backscatter diffraction (EBSD) detector (e.g.,^27^) was performed on two samples; one from the damage zone and the other from the fault gouge to perform a semi-quantitative analysis. For the measurements in a vacuum (< 0.01 Pa), we used a high voltage of 18 kV and a current of 1.2 nA, at a working distance of 10 mm. The readout time was set to 10 s, with a points-per-time setting of 4. The results show that plagioclase, quartz and alkali feldspar are the dominating mineral phases in both samples. This information was derived by randomly targeting grains and analysing their compositions.

For convenience in subsequent experiments, the fraction of < 63 μm was separated using wet sieving. Then, the sample was heated in a furnace to 400 °C for 30 min in air to reset any remaining natural dose associated with temperatures below 400 °C and avoid mineral phase changes that may occur at higher temperatures. The sample was then irradiated with a gamma dose of 75 Gy at the Karlsruhe Institute of Technology (Germany), resulting in an absorbed dose of 71.50 ± 0.15 Gy, taking into account irradiation geometry and attenuation effects.

### Rotary shear experiments

**Fig. 2:**
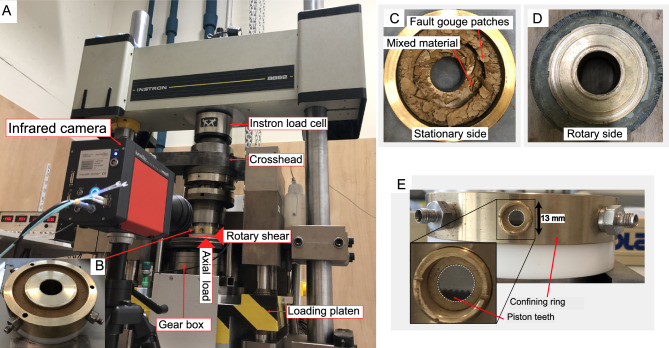
(**A**) Rotary shear apparatus equipped with an infrared camera. (**B**) The powdered material inside the specimen chamber before the experiment. (**C**) The top view of the gouge sample after the friction experiment, and (**D**) the rotary side. (**E**) The confining ring, the piston teeth and the transparent window (with the white dashed circle) are marked. The white-dashed frame shows the camera zoomed in on the region used to record the thermal images shown in Fig. 3A–C. Photos of (**A**–**D**) were taken from an unirradiated fraction of the gouge sample to illustrate the experimental setting.

To simulate slip in fault gouges, we performed a medium-velocity friction experiment on the irradiated material using the rotary shear apparatus^28^ at the Earth Simulation Laboratory at Utrecht University (Netherlands). The apparatus consists of a Parker MH205 motor coupled to a 1:160 harmonic drive gearbox housed within a 100 kN Instron 8862 load frame, equipped with a servo-controlled electromechanical actuator (Fig. 2A). The motor drives a loading platen, which locks the sample assembly into place. The sample assembly consists of a pair of stainless-steel rings (100 mm and 70 mm outside and inside diameter, respectively), which sandwich a layer of powdered material (“fault gouge”). The surface of the rings is machined with 0.7 mm-deep grooves, providing sufficient roughness to prevent slip at the interface. The gouge layer is confined laterally by an inner and an outer bronze ring. The outer ring houses a 2 mm thick sapphire window with a 10 mm diameter. In each experiment, a layer of ~ 3.5 mm thick was prepared in a dark room by depositing a total of 30 g of material on the bottom piston with the inner and outer confining rings in place. An aluminium spacer was used to distribute the material evenly, while checking with a level. The sample assembly was then closed by placing the top piston over the gouge layer.

Here, we present the results of three experiments under room-dry conditions. The first and second experiments were identical in terms of boundary conditions: a normal stress of 12 MPa, a slip velocity of 0.05 m/s, and a slip duration of 40 s. The last experiment used the same setting, though the device stopped after 18 s due to a failure of the motor control software. The applied normal stress is equivalent to that expected at depths of 0.5–1 km, depending on faulting style and pore pressure^29,30^. The imposed slip of ~ 2 m is equivalent to an earthquake of ~ M 7.0, albeit with a lower peak slip velocity^1^.

After running the experiments, we aimed to separate the extremely localised slip layer (i.e. Principal Slip Zone, PSZ) from other layers that experienced less slip (i.e. weakly deformed gouge) during rotation. This step was not straightforward, as at first glance, the entire material appeared mixed. However, the highly localised slip patches could still be distinguished due to their white (light) colour under subdued red-light conditions. We also separated some patches that were not evidently associated with the PSZ or the stationary side and named these mixed materials (weakly deformed gouge) (Fig. 2C).

### Temperature monitoring

The mechanical work during a co-seismic event is primarily partitioned into frictional heat and surface energy, with possible contributions from radiated and stored elastic energies^21,31^. Assuming the latter is negligible in our experiment, the mechanical work is fully converted to heat. To record the heat distribution and evolution in the gouge zone, we utilised a high-speed infrared (IR) camera for recording the temperature rise during experiments^32^. The increase in temperature plays a pivotal role in resetting the luminescence signal in fault gouges during earthquakes. As such, combining friction experiments with an infrared camera helps estimate the potential degree of luminescence signal resetting by recording temperature, its temporal evolution, and its spatial distribution across the sample.

The IR images were recorded through a circular sapphire window at the outer part of the confining ring (Fig. 2E) using an Infratec ImageIR 8300 hp with a precision microscope lens M = 1.0x, recording full frames at 200 Hz to map the IR signature across the gouge zone within the sample chamber for each of the three friction experiments. The IR signatures are converted to temperature, assuming the default values for IR emissivity (0.91) using the IRBIS^®^ 3.1 Infrared Thermographic Software. With this setting, we assume the radiance contribution from the polished stainless steel and brass components of the shear-ring apparatus to be negligible. The camera’s setup, in conjunction with the rotary shear, was developed by Hung^28^.

### Luminescence signal measurements

The experiments produced material which we divided into the PSZ and mixed material (a combination of weakly deformed zones). Since the conditions of the first and second experiments were identical, the fault gouges extracted from the first (FGA) and the second (FGB) experiments were combined and named FGAB; the mixed material was named MMAB. The fault gouge of the last experiment, FGC, was kept separate. Additionally, we retained irradiated material that was not subjected to any friction experiments (henceforth, standard material, SM). In total, four subsamples were prepared for luminescence analysis following common treatments to extract the polymineral fine-grain (4–11 µm) fraction, including the removal of organic (H_2_O_2_ treatment) and carbonate (HCl treatment) components, and, lastly, clay minerals through gravimetric separation in settling cylinders.

Our experiments commenced with a routine approach using infrared stimulated luminescence (IRSL^33^) and post-infrared stimulated luminescence at 225 °C (pIRIRSL_225_)^34^ measurements on a Freiberg Instruments lexsyg SMART TL/OSL system^35^ in the blue/violet wavelength range. Unfortunately, we were unable to measure any signal for the SM sample (data not shown). Therefore, we moved on to TL as a direct measure of the temperature-induced signal reset. These TL measurements were conducted using a Freiberg Instruments lexsyg research reader^36^ at Heidelberg University. The reader was equipped with a ^90^Sr/^90^Y ring source^37^ and delivered 0.256 ± 0.004 Gy/s on fine grains (4–11 µm at calibration date, 2024-05-13, quartz fine grain, #3Gy_0116.3 ± 0.03 Gy;^38^). TL signals were detected with a Hamamatsu H7360-02, through a Schott BG39 (1 mm), and a Chroma 410/40 ET filter. We applied a nitrogen atmosphere and a heating ramp of 2 K/s. Each TL measurement was followed by an immediate background measurement with identical settings to obtain the net TL signal through channel-wise subtraction. No preheat was applied.

We investigated raw TL signals measured at either 450 °C or 600 °C and followed the multiple-aliquot additive-dose (MAAD; first described by^39^, also^40^) approach to determine each sample’s equivalent dose (*D*_*e*_). In the MAAD approach, each sample is split into a suite of aliquots treated separately. The TL readout is performed only once per aliquot. The active cooling temperature was set to 25 °C before each new step. Athermal fading^41^ was not considered, as all samples were compared to our reference sample SM, which underwent identical procedures except for the rotary shear experiments. In other words, we compare relatively to the sample SM that was not further processed in the friction experiment.

For the MAAD approach (TL to 450 °C), we prepared six aliquots for each additive dose point for our four samples (SM, FGAB, FGC, and MMAB); 24 in total per sub-sample. Sample SM was the reference that should ideally reproduce the given gamma dose. The first set of aliquots of each sample was treated as natural and received no dose (zero dose). Other sets received the following additive beta-dose before TL readout: 76 Gy (± 1.5%), 152 Gy (± 1.5%), and 305 Gy (± 1.5%). After one month, the experiment was repeated to increase the statistics. In the repetition, the experimental conditions were almost identical, except for the time between administering the additive dose and the TL readout, which was 12 days (initial measurement) and 18 days (repetition). For subsequent analysis, results from both experiments were combined, and the *D*_*e*_ was calculated by extrapolation.

The data analysis was carried out using the statistical programming language R^42^, packages ‘ggplot2’^43^ and ‘Luminescence’^44^. For the analysis of the net TL signals in the MAAD approach, we identified the peak of interest manually below ca. 250 °C. Then we integrated counts over varying temperature ranges (with the identified peak centred around 160–180 °C) for comparison. We used the standard deviation of the initial 40–52 °C (n > 50 channels) of each TL measurement to derive uncertainties from the counting statistics.

The aliquots used for the MAAD analysis were later used again for spectrometer measurements. The spectrometer measurement settings followed those of the TL measurements, except that higher doses were applied to compensate for the spectrometer system’s significantly lower detection efficiency compared to the photomultiplier tube (PMT) measurements. Doses administered through the in-built beta source were 250 Gy, 750 Gy, 15 kGy, and 60 kGy (relative irradiation uncertainties: ± 1.5%).

The spectrometry measurements were carried out to better understand the TL signal behaviour of our rock samples before and after the friction experiments. The measurements were performed on the same machine connected with an optical fibre to a Shamrock (Andor/Oxford Instruments) SR-163 spectrometer. The light was detected using an Andor Newton DU920P-BEX2DD CCD camera with a 300 l/mm grating (blaze: 500 nm) and the entry slit fully open. The equipment was wavelength calibrated. We approximated the detection efficiency using transmission data tabulated by the manufacturer of silica windows, optical fibre, grating, and camera. No hardware binning was applied. Signals were integrated over 1.8 s with a channel length of 2 s. Detectable wavelengths for our spectrometer settings ranged from 248 to 752 nm. All spectrometer measurements were subject to cosmic-ray removal before analysis and visualisation using available functions from the R package ‘Luminescence’. The visualisation settings (e.g., additional binning) were chosen to improve the quality of the figures.

## Results

### Frictional heat and temperature rise

The infrared camera produced continuous thermographic images during friction experiments, demonstrating the evolution of temperature rise along the IR-transparent window.

**Fig. 3:**
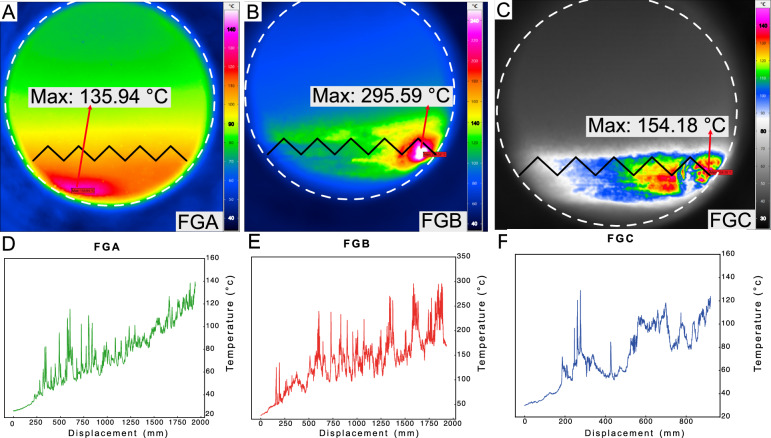
(**A**–**C**) Infrared thermographic images of the three friction experiments of our study. (**A**), (**B**) and (**C**) illustrate the highest temperature for each setting. The white dashed circle and piston teeth are associated with Fig. 2E. The highest temperature is produced on the interface between the piston teeth and the gouge sample. (**D**–**F**) The evolution of maximum temperature with displacement during friction experiments is shown for three samples.

The highest temperature recorded by the camera is marked for each experiment in Fig. 3A–C. Although the setups for FGA and FGB were identical, the highest temperatures recorded differed: FGB showed significantly higher temperatures than FGA. The temperature of 296 °C is high and could fully reset the investigated TL signal. However, as shown in Fig. 3B, the temperature distribution is highly heterogeneous, and the highest temperature appears to be confined to the extreme slip interface on the right-hand side of the image. This relatively high-temperature area overlaps with the location of the rotating stainless-steel piston (Fig. 3D). Nonetheless, the gouge layer at the piston interface or at the tooth contact points exhibited temperatures limited to ca. 200 °C.

Figure 3A shows a more homogeneous temperature distribution than Fig. 3B. However, the highest temperature recorded is limited to 136 °C and is located at the bottom of the sapphire window, specifically at the location of the rotating piston. At the interface, the temperature does not exceed ca. 110 °C. Still, it shows a much more homogeneous temperature at the sliding surface than Fig. 3B. The overall temperature at each image in Fig. 3A–C, further away from the interface, is homogeneous and remains at 80 °C for both Fig. 3A,B.

Figure 3C shows two distinct clusters with higher temperatures than other areas across the frame. The highest temperature is 154 °C, but temperatures above approximately 130 °C are observed at several small patches. The temperature is not homogeneous; above the interface, it is limited to 40 °C. The maximum temperature evolution over displacement for each experiment is depicted in Fig. 3D–F, where it is evident that the temperature does not increase linearly over time during the friction experiments, and the temperature rise is subject to fluctuations and spikes. For instance, in Fig. 3E, the temperature drops from 296 to 248 °C before rising again to 294 °C.

Our measurements indicate that the highest temperature occurs at the interface between the gouge and the rotating piston, within a zone less than 800 µm wide. However, it is highly localised, and we infer that even submillimetre tilts of the piston cause a highly heterogeneous temperature distribution in our experiments. This is further complicated by the extrusion of material into the narrow gap between the piston and the sapphire window.

### Thermoluminescence signal and spectrometry results

**Fig. 4:**
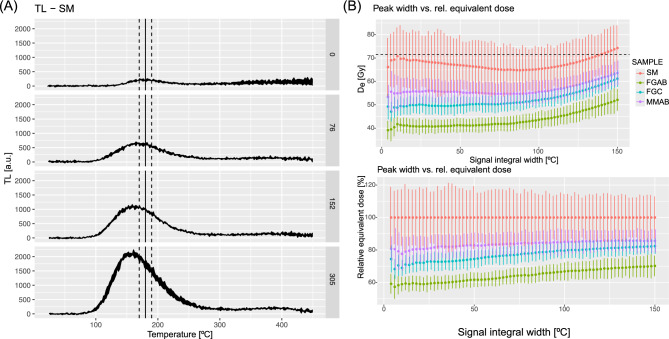
Results of the MAAD experiments. (**A**) Additive net TL curves (top to bottom) for the natural SM and irradiated aliquots. The zero dose corresponds to the TL signal from gamma radiation. The doses of 76 Gy, 152 Gy, and 305 Gy were delivered to the aliquots in sequence. Each row in the graph shows different additive doses. The investigated TL peak is marked with a solid vertical line, and the signal integrals are marked with dashed lines (here ± 10 °C). (**B**) Equivalent dose results are a function of the chosen peak width; peak centre as indicated in (**A**). The upper plot shows absolute dose values, and the lower plot shows the relative signal reduction. The dashed horizontal line indicates the administered gamma dose.

Figure 4 depicts the results of the MAAD experiments. Figure 4A shows the net TL readouts of the sample SM after additive doses of 0 Gy (natural), 76 Gy, 152 Gy, and 305 Gy. For better visualisation, the curves display the TL signal range (i.e., the signal range of all measured TL curves for each dose), whereas the analysis was performed on individual curves. Figure 4A shows that the obtained signal is barely distinguishable from the background, with a peak located at around 180 °C. This peak shifts slightly towards lower temperatures (ca. 160 °C) as the administering laboratory dose increases, indicating the presence of a thermally unstable signal regardless of the waiting time between additive dose irradiation and measurements. The same pattern was observed for all samples.

Nevertheless, the TL curves are remarkably reproducible across all samples, except for a few aliquots that exhibited spurious, very bright signals, likely due to contamination. We removed those aliquots from the analysis. Because of the complex TL peak structure, we ran our analysis with different peak integration limits (Fig. 4B) while maintaining a fixed peak centre at 180 °C. Overall, the *D*_*e*_ of the treated samples (FGAB, FGC, and MMAB) is at least ca. 20% lower than SM. The highest reduction is observed in sample FGAB, and the lowest in the mixed sample MMAB. Graphical output from all individual TL and corresponding dose–response curve fitting can be found at Zenodo^45^. The reduction is greater with narrower peak-integral settings. Regarding the question of which signal integral is most trusted, the answer would be unsatisfactory without TL peak deconvolution, which we attempted but discarded due to the low signal intensities and related uncertainties.

**Fig. 5:**
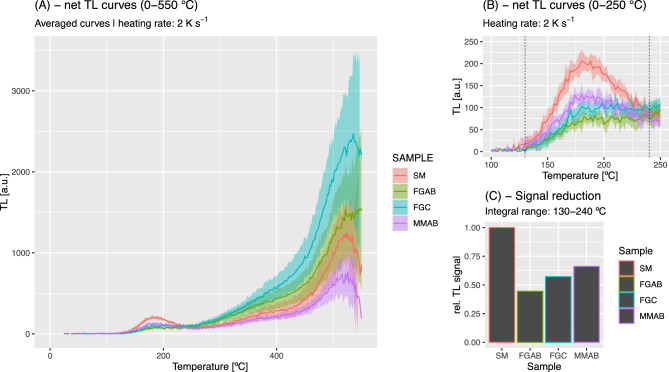
TL readouts of samples SM, FGAB, FGC, MMAB. (**A**) Net TL curves from all samples recorded to 600 °C (for visualisation only shown to 550 °C). The solid lines are the average of a minimum of five curves. The polygon shows the standard deviation of the five curves for each subsample. (**B**) Close-up of the peak at 180 °C with the chosen signal integral indicated by vertical dashed lines. (**C**) Barplot of the relative signal reduction deduced from the signal integral in (**B**).

However, the high reproducibility of the TL signal from individual aliquots enabled us to run another experiment using the non-friction-affected TL signal as a reference to determine the reduction caused by frictional heat from the differences in count values alone. In this experiment, all TL curves were recorded up to 600 °C. The results are illustrated in Fig. 5A–C. Using the TL signal integral around the 180 °C peak (Fig. 5B), the pattern established in Fig. 4 remains, but the results appear more accurate because they are not biased by overlapping peaks in a complex peak structure. The highest signal reduction was observed in sample FGAB, which showed ca. 55% of the signal of sample SM, followed by sample FGC (ca. 45% signal reduction) and sample MMAB (ca. 35% reduction). No complete signal reset was observed, and the signal pattern above 250 °C is exhibited in Fig. 5A. Although signal scatter increases with temperature due to blackbody radiation that increasingly superimposes on the TL signal of interest, a peak around 520 °C appears to have been enhanced by the friction experiments, and this pattern seems to correlate slightly with the degree of signal resetting.

**Fig. 6:**
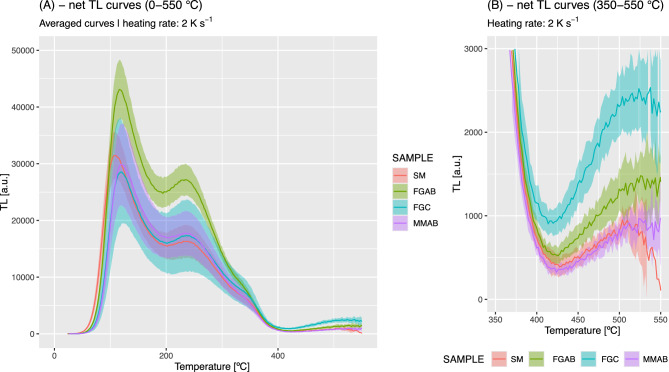
Regenerated TL readouts of samples SM, FGAB, FGC, and MMAB following the experiments in Fig. 5 and after a dose of 71.50 ± 0.15 Gy. (**A**) The whole curve is displayed up to 550 °C, and (**B**) shows only the temperature range between 350 and 550 °C for better illustration.

The drawback of the TL readouts at high temperatures, such as 600 °C, and therefore we initially avoided them for signal resetting, is that the aliquots likely exhibit different signal patterns afterwards due to possible phase transitions in the polymineral fraction, such as reported for quartz (e.g.,^46^) and feldspar (e.g.,^47^). For instance, in quartz, this significantly increases its radiation sensitivity (e.g.,^48^). Indeed, Fig. 6A,B exhibit a considerable increase in luminescence sensitivity by a couple of orders of magnitude in the investigated wavelength range. Whether the enhanced intensity can be attributed solely to quartz is unknown and could not be further investigated due to limited material availability. Strikingly, however, the established signal pattern from the friction experiments persists for the peak around 520 °C. The results in Fig. 5 indicate that the signal reset and, with it, the temperature reset by frictional heat seem to have affected only the low-temperature peaks (< 200 °C).

### Thermoluminescence spectrometry measurements

**Fig. 7:**
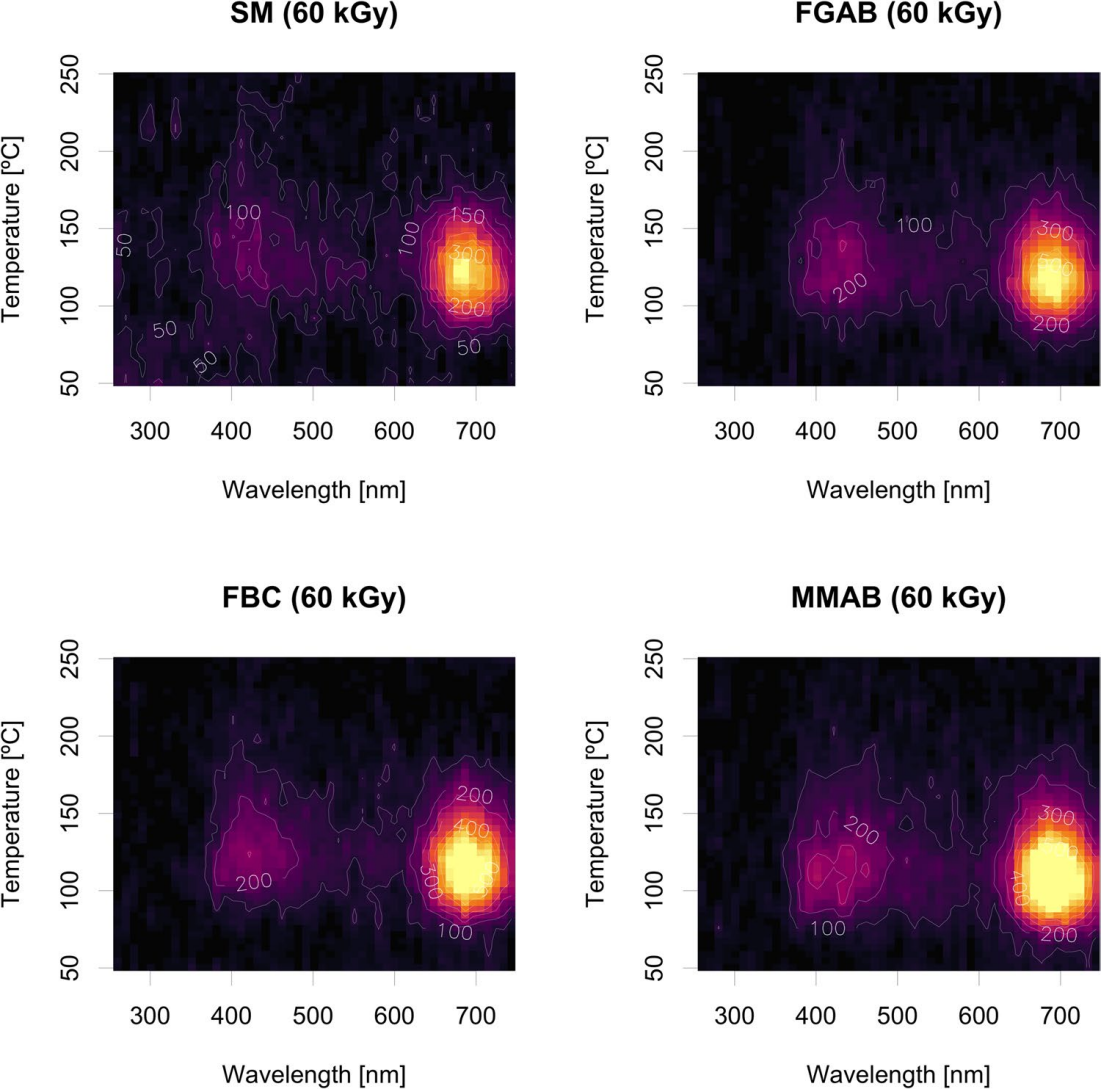
TL spectra of four samples after receiving the dose of 60 kGy. The focus is on peaks below 250 °C, as we observed no distinguishable peaks between 250 and 450 °C, the highest temperature for the spectrometer measurements. The spectra of all four samples are relatively dim, with low-temperature TL peaks located between ca. 380 and 435 nm and ca. 670 to 720 nm. Note that the difference in the peak position compared to the TL experiments is related to the pause between irradiation and signal readout. Prompt readouts were used for the spectrometer measurements due to limited machine time.

As shown above, TL signals were generally dim for all samples. Therefore, we administered doses up to 60 kGy before our spectrometer measurements. Still, the spectra of SM’s natural and irradiated aliquots remained very weak across all samples, and no TL emission below 750 Gy and above the background could be measured with our system. Therefore, in Fig. 7, we present only the results after a dose of 60 kGy in the temperature range below 250 °C, as we could not detect measurable signals between 250 and 400 °C with our system, and above, as the signal was strongly masked by blackbody radiation (Fig. 7).

All samples exhibit similar low-temperature emission in the UV/violet/blue range from 380 to 435 nm, accompanied by a strong emission band centred around 670–720 nm. With increasing dose, another emission in the green wavelength range, around approximately 550 nm, appears to emerge. However, the spectrometer measurements did not reveal a change in the overall composition of the emission spectrum that might be caused by friction experiments, as observed in the TL curves.

## Discussion

### Temperature rise and signal resetting in friction experiments

TL signal resetting appears to occur to some degree in samples FGAB, FGC, and MMAB; however, it did not affect all polymineral samples uniformly. The thermographic images from the infrared camera (Fig. 3B) revealed that the approximate temperature required to reset the TL signal of interest fully had been reached, at least in a highly localised area. However, this seems to have occurred only in a tiny patch less than ca. 1 mm thick, consistent with field observations indicating that the “extremely localised” PSZ is only a few millimetres in thickness or even thinner^49^. While the overall available volume of material will be higher in nature, from a technical point of view, identifying and extracting this patch from natural fault rocks is challenging, and it seems to be the main obstacle to accurately dating fault gouges and to overestimating the timing of the last major earthquakes. It is worth noting that higher temperatures could be reached if a slip velocity of 1 m/s, as expected in earthquakes^50^, were applied under the same normal stress. The applied normal stress of 12 MPa would correspond to a depth of ~ 0.45 km (assuming a density of 2650 kg/m^3^) in the absence of fluid pressure or ~ 0.7 km with hydrostatic pore pressure, which could offset the temperature rise expected for surface-breaking earthquake ruptures where the normal stress is much lower (or in other words, 12 MPa and *v* = 0.05 m/s would generate similar heat as 0.6 MPa and 1 m/s, in the absence of rapid heat conduction).

We observed that the temperature rise in FGA and FGB did not agree with one another. In contrast, we expected the same temperature to be produced due to the similar experimental setup. Saber^51^ reported that slip localisation at the contact areas can vary to some extent in two different friction experiments, which could explain the difference in temperature mapping between FGA and FGB. Another possible explanation for this discrepancy is a non-homogeneous distribution of the gouge sample before the friction experiment. However, the difference in temperature rise for these two experiments is significant, making this explanation rather implausible. Instead, the observed temperature may result from extruded material getting stuck between the piston and the window. However, we did not observe significant differences in the evolution of the layer thickness between the experiments.

We mixed FGA and FGB to ensure sufficient material after the experiments. In addition to chemical treatments, the clay fraction was removed ahead of the luminescence measurements. This procedure resulted in mixed fault gouge patches, which, in hindsight, are likely to experience different temperature conditions during slip. In other words, the possibility of including fault gouge layers with various degrees of exposure to frictional heat cannot be ruled out. It is worth noting that selecting patches based on their general location (not taken from the stationary side), colour, and degree of grain size reduction, according to apparent texture (Fig. 2), particularly in a dark room, is challenging and requires more experience to optimise this procedure.

Another critical issue to consider is that, except for Fig. 3A, in which the highest temperature arises from the bottom of the piston, the heating appears to originate from the piston teeth instead of the gouge itself. These extremely localised slip layers are located very close to the slip surface (Fig. 3B,C). As the IR emissivity of the stainless steel is lower than that of our sample (dominated by quartz and feldspar grains), our observation could be explained by possible extrusion of material during slip, a common phenomenon in rotary shear experiments^19^. Friction between the extrusive materials and the sapphire window could have caused the high temperature recorded by the camera. However, in this case, the window should become visibility blind over time due to material scratching the window, but such an effect was not observed in our setup.

### TL curve shape patterns and spectrometry results

The TL curve shapes and absolute intensities, although dim, proved highly consistent across aliquots for the four samples. Given that we compared all findings to the sample SM, not subject to friction experiments, athermal fading was not a critical factor in our experiments. Potential signal loss due to different pause times between irradiation and measurement for two batches (12 days vs. 18 days) was not quantifiable.

Still, the results from the TL measurements in the violet/blue wavelength region were unexpected. First, given the thermal images from the rotary shear experiments, we expected to observe a complete reset for at least one of the sub-samples for the low-temperature TL peak investigated. Instead, we observed a reduction of ca. 55% (at most) relative to the reference signal for sample SM. Although this finding is disappointing, it is most likely related to the highly localised temperature distribution, which only occurs within a tiny patch of sample material that is difficult to separate after the experiment. This situation is expected to be less severe in nature, allowing a larger volume to be sampled. Furthermore, we conducted our experiments at a relatively low slip velocity due to limitations of our equipment. Hence, experiments with higher slip velocities are indispensable for future studies, but measuring the temperature distribution in such experiments will be even more challenging.

Secondly, we observed a change in TL signal intensity at temperatures above 250 °C for the samples most affected by friction. Given the similar treatment of all samples and the highly reproducible signal patterns across aliquots, the most probable explanation for this signal enhancement is friction. Notably, this change in signal intensity persists even at 600 °C (Fig. 6), with a phase transition of some minerals likely indicated in the TL curves, which are now dominated by the quartz signal. While fundamental studies such as those by McKeever et al.^52^ have already shown changes in the TL peak structure after temperatures up to 500 °C, in our case, the friction experiment seems to superimpose another effect. This observation, the persistence of the friction-related peak difference even after such high temperatures, is, to the best of our knowledge, new. It is probably related to our specific host rock mineral composition and cannot be easily transferred to other settings. However, given that the friction appears to be recorded within the sample material, this opens new possibilities for assessing the sample position in fault gouges that experienced the highest frictional heat. Further studies are needed to investigate this phenomenon more thoroughly.

A critical part of our experiment was our focus solely on a low-temperature TL signal, which we used as a proxy for resetting conditions during the friction process. Those signals are, regardless of athermal fading, thermally unstable on time scales relevant to palaeoseismology. For such studies, signals above 300 °C are usually analysed. In our case, the natural sample, however, was not bright enough to study those signal peaks. Moreover, the aforementioned signal enhancement is likely to alter the outcome of such an investigation, and the required experiments to correct this feature are beyond the scope of our current study. For transparency, we also investigated the red TL emission low-temperature peak and performed preliminary infrared radiofluorescence (IR-RF^53,54^) measurements. However, those measurements did not yield any new information, and the results were neither discussed nor included in our article.

Feldspar luminescence spectra are more complex than quartz luminescence spectra due to differences in crystal structure and chemical composition^55,56^. In this study, we measured the TL spectra of a polymineral sample from the volcanoclastic rock of the Alborz region, which is mainly composed of alkali feldspar, plagioclase, and quartz. This further complicated the spectral composition. Surprisingly, the spectra of all samples, even after 60 kGy irradiation, remained dim, despite the considerably lower detection efficiency of the spectrometer setup compared to that of the PMT measurements. On top of the low efficiency of our setup, this might be explained by the origin of our samples, which were taken from the volcanic environment of the Alborz provenance, as^55,56^ highlighted that the luminescence signal of volcanic material tends to be dim.

Krbetschek et al.^57^ also reported that the blue-violet emission of the TL signal obtained from polymineral fine-grain spectra was generally associated with feldspar. However, quartz can also contribute to this emission. K-rich feldspar has a strong emission at violet-blue or blue band (ca. 400–420 nm, 425–480 nm) while the Na-rich feldspar emits at 560 nm or a general range of 500–600 nm (green-yellow) Krbetschek et al.^57^. These reports align with the emissions from our samples, which exhibit two peaks at wavelengths between 380 and 435 nm. Although only a faint glow is observable at around 550 nm, which does not constitute a clear peak, it is likely related to our setup and requires further optimisation for future measurements.

## Conclusion

In this study, we presented friction experiments performed at medium velocity using a rock sample from the NTF zone of the Karaj Formation of the Alborz Mountains. A slip velocity of 0.05 m/s combined with a normal stress of 12 MPa was employed in this study to investigate whether the TL signal of a polymineral fault rock sample is reset during friction experiments. We did not use any chemical treatment before our experiments, and a powdered version of the intact rock was employed in the friction experiments after being reset and irradiated with a known gamma dose.

We used an infrared camera to monitor the spatial evolution of temperature during slip and found that the temperature distribution is heterogeneous and highly localised. We demonstrated that a slip velocity of 0.05 m/s, combined with a normal stress of 12 MPa, produced sufficient heat, resulting in, at a minimum, a partial reset of the TL signal.

The thermal images showed a maximum temperature of 296 °C in our experiments, which should be sufficient to fully reset the investigated TL signal. This means that an earthquake with slip velocities of ca. 1 m/s or higher could reset the fault gouge near the Earth’s surface (0.5 km). Thus, fault gouge dating has the potential to provide a reliable estimate of the timing of past major earthquakes. 

However, the main drawback of this method is the technical limitation of identifying and extracting the extremely localised slip layer, which experiences the highest frictional heating and is accordingly heated to a sufficiently high temperature. Without this identification, fault-gouge dating yields an overestimated age.

This was the first time the TL signal of a polymineral rock sample was shown to be reset during a laboratory friction experiment, and we found that the friction experiment enhanced the TL signal. This aspect will require further attention in follow-up studies.

TL spectrometry of the rock sample from the Karaj Formation exhibited a very weak signal, and the spectrum could be measured only after 60 kGy. Although no infrared-stimulated luminescence signal could be measured at low or high temperatures, we successfully recovered the given dose using the TL signal of our polymineral fine-grain sample.

## Data Availability

Raw and partially processed data are available at Zenodo: 10.5281/zenodo.16904960
